# Indecision on the use of artificial intelligence in healthcare—A qualitative study of patient perspectives on trust, responsibility and self-determination using AI-CDSS

**DOI:** 10.1177/20552076251339522

**Published:** 2025-05-30

**Authors:** Diana Schneider, Wenke Liedtke, Andrea Diana Klausen, Myriam Lipprandt, Florian Funer, Tanja Bratan, Nils B. Heyen, Heike Aichinger, Martin Langanke

**Affiliations:** 1 Department of Emerging Technologies, 28479 Fraunhofer Institute for Systems and Innovation Research ISI, Karlsruhe, Germany; 2 Faculty of Theology, University of Greifswald, Greifswald, Germany; 3 Department of Social Work, Protestant University of Applied Sciences Rhineland-Westphalia-Lippe, Bochum, Germany; 4 Institute for Medical Informatics, University Medical Center—RWTH Aachen, Aachen, Germany; 5 Health Services Research, 11233 Carl von Ossietzky University of Oldenburg, Oldenburg, Germany; 6 Institute for Ethics, History and Philosophy of Medicine Hannover, 9177 Hannover Medical School (MHH), Hannover, Germany; 7 Institute for Ethics and History of Medicine, Eberhard Karls University Tuebingen, Tuebingen, Germany

**Keywords:** Responsibility, trust, self-determination, patients, artificial intelligence, clinical decision support systems

## Abstract

**Background:**

Patients are confronted with the digital transformation of medicine, yet there is a paucity of studies that discuss the patient perspective on AI-based clinical decision support systems (AI-CDSS). Our study addresses this research gap by focusing on their needs and concerns, especially regarding trust, responsibility, and self-determination.

**Methods:**

The qualitative study was conducted between April 2021 and April 2022 with 18 patients from Germany to participate in three focus groups (5–7 per group). The groups were presented with AI-CDSS examples (surgery, nephrology, and home-ventilated care) to discuss ethical and social aspects of their implementation. The interviews were analyzed in accordance with the structured qualitative content analysis of Kuckartz and Rädiker (2022).

**Results:**

The interviewees expressed considerable uncertainty regarding the AI-CDSS implementation. The results highlight the patients’ perspective of AI-CDSS as a supportive tool or as a second opinion, which could challenge long-held values of trust, responsibility, and self-determination, particularly the relationship between trust and responsibility may undergo a transformation, leading to a loss of both.

**Conclusion:**

The findings demonstrate that patients perceive the AI-CDSS implementation as a challenge both for their own decision-making and for the future doctor-patient relationship. This is further indicated by the shifts of trust, responsibility, and self-determination as influencing decision-making factors. Concurrently, the findings show that the patients’ perspective is profoundly influenced by the individuals’ comprehension of the functionality of AI-CDSS. It is therefore of importance to provide patients and healthcare professionals with information to prevent indecision.

## Introduction

The digital transformation in medicine has raised many hopes, especially as data can now be used to generate new insights that were not possible before. Among these technologies, methods of artificial intelligence (AI) such as machine learning have gained increasing prominence.^1^

AI-based clinical decision support systems (AI-CDSS) aim to assist healthcare professionals and patients in making decisions, particularly regarding personalized diagnoses and treatments.^2,3^ They are being used in various medical disciplines, for instance, radiology,^4^ dermatology,^5^ or surgery.^6^ Benefits include personalized diagnoses and treatment options, improvements in workflow structuring, coping with workforce shortages, and reducing medical errors.^1,2,7^

Alongside these significant potentials, ethical questions arise. Rajpurkar et al. state that various concerns like data security, responsibility, fairness and autonomy are being discussed, yet remain unsolved.^7^ Likewise, Amann et al. observe the challenges of losing explainability of AI in light of various stakeholders, for instance regarding the professional-patient relationship, as analyses and recommendations generated by the system that can no longer be understood by the users could lead to a potential new paternalistic movement and thus have a negative impact on the professional-patient relationship.^8^ As argued by Obermeyer and Topol, algorithms should thus be developed with a wider spectrum of stakeholders than just the doctor to ensure that the people affected by the system's analysis can also understand the system's results.^9^ Special consideration must be given to the AI-CDSS implementation from all users’ perspectives. This affects healthcare professionals,^10^ patients^11^ and especially the relationship between them.^12,13^ Yet Sauerbrei et al. state that while AI can be a disruptor of the healthcare system, only a few studies focus on the effects on the doctor-patient relationship.^14^ Still being mostly passive users, patients are the primary beneficiaries of AI-CDSS in these relationships, therefore respecting their interests and values is of utmost importance.^15^

Comparatively little research has been done on their perspectives on using AI methods in diagnosis and treatment. Only a few studies provide a deeper patients’ perspectives on the use of machine learning^16,17^ and even fewer address actual engagement in the context of AI-CDSS.^18^ Although, according to Madanian et al., a patient-centric approach is needed to address their needs and experiences in order to design and develop usable tools,^18^ most studies focus on questions of patient acceptance or general attitudes towards the usage of AI in specific disciplinary aspects like radiology,^19,20^ ophthalmology,^21^ dentistry^22^ or women's health^23,24^ or towards selected groups.

These rather general studies demonstrate different perspectives of patients regarding their take on the implementation of AI. AI technologies are viewed positively by patients in the context of structural assessment, e.g., with respect to waiting times and time to diagnosis.^21,25–27^ The optimization of structural processes in the context of supply management and the coordination of professionals are considered as a positive outcome.^26,28^ Meanwhile, patients are reserved when it comes to personal risks. Of particular concern is the deterioration of personal relationships with immediate healthcare professionals, a possible loss of human interaction in the entire care process, aspects of data security, the reliability of technology and possible risks of discriminating algorithms,^26,28,29^ a dehumanization and abandonment to “machines.”^28^

It is noteworthy that these studies rarely discuss the specific ethical challenges, that display conflicts of values, interests and needs of AI-CDSS implementation from the patients’ perspective. Although key aspects such as responsibility,^15,30^ justice^15^ and the professional-patient relationship^15,24,29,30^ are mentioned, a thematic exploration through the patients’ perspective is rare. Jeyakumar et al. as well as Vallès-Peris et al. highlight the integration of AI technology into treatment while considering patients’ values and needs to ensure a good professional-patient relationship.^15,30^ In greater depth, Dlugath et al. examine how patients envision a trustworthy AI, emphasizing on values such as fairness and reliability.^31^

This explorative study therefore focuses on the patient as part of the professional-patient relationship and patients’ perspectives and attitudes on the potential implementation of AI technology. Unlike previous studies that primarily catalogued various patient concerns regarding acceptance of AI in healthcare applications, our study aims to delve deeper into the ethical implications of these perspectives, specifically examining the challenges related to responsibility, trust and self-determination. These aspects were highlighted by the respondents as elementary for the provider-patient relationship. Although these aspects were frequently mentioned by patients in studies aiming to understand their perspectives and reservations, they were not deeply explored.^15,16,19,21,26,28,29^ With our explanatory approach, we fill the gap by presenting the aspects of responsibility, trust, and self-determination from the patients’ perspective and their intuitive understanding.

In academic discourse responsibility and trust can both be considered as relational concepts of different relata. There are discussions on the differentiation of the relata of responsibility^32,33^ and trust.^34,35^ Much of the discourse on responsibility in AI technology focuses on the subject,^3^ the normative implications and their prerequisites.^36^ However, trust is often discussed in terms of its quality of connection due to the understanding of the relationship between humans and machines. In particular, the question arises whether this relationship can be associated with trust^37^ or whether it would be more appropriate to speak of reliance in this context.^38^ In contrast, the discourse on self-determination, often equated with autonomy, focuses on the possibilities and abilities to make decisions independently and without coercion for one's self.^39,40^ Against the backdrop of AI technologies, this appears to be challenging due to an “AI paternalism.”^41^

While there are different concepts of responsibility, trust and self-determination, our empirical study was not primarily focused on a specific concept of these aspects. Rather we want to explore the intuitive patient's perception of these three aspects and examine them in relation to the implementation of AI-technology. Our study underscores that the perception of CDSS within professional-patient communication is critical to determining whether this relationship—particularly regarding responsibility, trust and self-determination—remains stable or is at risk of being significantly disrupted by the implementation of CDSS.

## Methods

The explorative study was created as part of the DESIREE project focusing on ethical, social, technical and professional aspects of implementing AI-CDSS into healthcare. The study is reported following the consolidated criteria for reporting qualitative research (COREQ).^42^ We conducted three focus groups with 18 patients (5–7 per group) between April 2021 and April 2022. Informed consent of the participants was obtained prior to the empirical study in written form. The focus groups aimed to provide a communicative space in which patients could exchange opinions and viewpoints as experts in homogeneous groups.^43^ Following Przyborski and Riegler, who see focus groups as an expression of collective knowledge, we use focus groups to determine whether and where shared experiences exist.^43^

Convenience sampling was performed. Participants were patients ≥ 18 years of age, had sufficient knowledge of German, were able to consent, and were self-reported to be in sufficiently good health. Acquisition took place with the help of self-help groups. Disease-specific self-help groups that provided peer-to-peer support for a particular condition in surgery, home care, and nephrology, and were eligible for participation in the study were contacted. The disease-specific self-help groups forwarded the invitation to participate in the study to their members, who could register for the study on their own initiative. Apart from one participant (acute hospitalization), all people recruited for the study took part in the focus groups.

The study was developed by an interdisciplinary team consisting of ethicists, sociologists, biotechnologists, medical informatics specialists, medical and nursing professionals, and health scientists. The team consisted of three men and six women. The empirical study was conducted by junior and senior researchers, who were trained in qualitative methods of social research and have many years of experience. No relationship was established between participants and researchers prior to the focus groups. The respondents were informed about the reasons and interests in the research topic as well as about the institutional affiliation of the participating researchers. In some cases, focus group participants knew each other due to their self-help group engagement. Respondents were told in advance only that the interview topic was “Clinical Decision Support Systems and Digitization in Healthcare.” Respondents received a small amount of financial compensation for participating.

Due to the COVID-19 pandemic and mobility issues of some patients, all focus groups were conducted via video call. Respondents were generally at home during the focus groups; most respondents were alone unless they needed assistance of a caregiver. However, caregivers did not actively participate. Interviewers used interview guides (see Supplement). Due to the explorative approach of the study, three very different fields of application (nephrology, surgery, and home care) were selected to elicit the most contrasting patient perspectives as possible. Within each focus group, CDSS case vignettes including illustrations that presented three different CDSS case scenarios were used as stimuli. The foundation for the vignettes was developed within the interdisciplinary research team.^44^ The three CDSS case vignettes were inspired by actual CDSS tools under development: (1) the AI-assisted “CKDNApp” to assist nephrologists in prognostic assessment and therapy planning for patients with chronic kidney disease^45^; (2) an AI-CDSS similar to the Da-Vinci robot^46^ to assist surgeons in intraoperative navigation and by providing recommendations for incisions to be made; and (3) the non-AI-based “Safety Box,” an alert system, similar to an AI-CDSS, designed to provide caregivers and family members with recommended actions for (emergency) care of ventilated patients in the home setting, similar to MeSiB.^47^ The CDSS case vignette (3) was developed further to fulfill the requirements of an AI-based CDSS. Per focus group, one of these CDSS examples was presented. Afterwards, respondents were asked about their thoughts regarding the implementation of this technology, particularly with regard to the relationship between healthcare professionals and patients.

Audio recordings were made of the focus groups and these recordings were transcribed ad verbatim and pseudonymized. Field notes were taken during and after the focus groups. The data collection was concluded when theoretical saturation was achieved, meaning that additional focus groups would not yield new information regarding the research question.

The focus groups lasted an average of 114:17 min (range between 110:07 to 123:04 min), including a short break after one hour. 18 people were interviewed (see Table 1): twelve of the interviewees described themselves as women and six as men. 10 people had completed high school and/or college. Respondents ranged in age from 24 to 76 (average 45.5).

**Table 1.** Sociodemographic data of patients participating in focus groups.

	Focus group nephrology (N)	Focus group surgery (S)	Focus group home ventilated care (H)
Quantity	5	6	7
Average age (age range)	47.8 years (24–76 years)	51.8 years (26–62 years, 1 unknown)	39.4 years (25–58 years)
Gender (self-reported)	4 ♀ / 1 ♂	3 ♀ / 3 ♂	5 ♀ / 2 ♂

Data analysis was performed using structured qualitative content analysis according to Kuckartz and Rädiker.^48^ Categories were assigned to the writings, which were derived both deductively from the literature review and inductively from the focus groups themselves. Two of the authors [DS, WL] independently coded the collected data using MAXQDA 2020, and unclear passages were discussed within the research team and subsequently resolved by consensus. Coding and analysis focused on specific themes such as responsibility, trust, and self-determination. Relevant quotes were selected to answer the research question. Original statements are translated into English for publication; long quotations are paraphrased.

## Results

The empirical results on trust, responsibility, and self-determination will be presented in the following three sub-chapters. While patients also imagine settings in which they could interact intensively with AI-CDSS, our focus will be on the conventional setting featuring active healthcare professionals and receiving patients.^3^ To categorize the patients’ original statements from the application-specific focus groups, we use the abbreviations “N” for nephrology, “S” for surgery, and “H” for home ventilated care.

### Trust

Patients expect that using AI-CDSS could have an impact on the professional-patient relationship, particularly on aspects of trust. They anticipate that using AI-CDSS could contribute to an increased level of trust and a sense of security, as professional skills could be trained through repeated AI support (S126); medical or nursing staff could be relieved given the existing shortage of skilled workers (H80); or new staff could be trained better (H153). Occasionally, they pointed out that AI-CDSS could be used in patient education (N34, H137). Yet patients also voiced concern that AI-CDSS could be preferred for economic reasons (H58, 80) and treatment data could be in the hands of unauthorized third parties (H56, 58, N61).

Indeed, interviewees characterized the relationship between medical or nursing professionals and patients and their relatives with the attribute of trust. The respondents named three elements that promote a trusting relationship: Firstly, patients emphasize that they trust professionals because of their education: “the doctor who has completed his studies, who has learned this for years” (N79).

Secondly, patients’ trust is associated with the professional's practical experience. The number of procedures performed by the healthcare professional is seen as a guarantee of competence. While a person with little professional experience, a “newbie” (N67), is given less trust than a person who has already carried out “around 800 colonoscopies” (S44). This experience includes challenges in practical work and dealing and communicating with patients (N77).

Last but not least, patients vaguely refer to a gut feeling in order to characterize trust as a leap of faith in the professional: “So in the end, I think it's the gut feeling and the doctor who has years of experience” (N83). In contrast to the previous, more objective criteria of education and experience, this is a subjective, more personal component. The basic consideration for patients seems to be the trustworthiness of a person on an interpersonal and/or moral level.

Only one person describes trust in a professional-patient relationship as having no alternative and emphasizes that patients have to trust the professionals because they are at their mercy in case of doubt: “Either way, I have to trust my surgeon and there's nothing I can do anyway” (S122).

The characteristics of trusting relationships are transferred by patients to the interaction of professionals with AI-CDSS: Similarly to the specialist knowledge of the professionals, patients emphasize the proven performance of AI-CDSS, e.g., better data analyzes through “results based on mathematical formulae” (N85, also H74) and better results when performing surgery: “as a patient, I am primarily interested in the results” (S136). By expecting better results using CDSS, patients appear to attribute expertise and practical experience to the system that is similar to that of a competent professional. However, patients also point out that statistical results cannot necessarily be transferred to individual cases (N63), therefore causing uncertainty among them.

When AI-based CDSS are used in practice, patients expect that healthcare professionals are appropriately trained and that they are in favor of using the system:

Because I think that if the doctor offers this, then he has also received a certain amount of training on this part […], so I assume […] that he is then also sure, the surgeon, that the system also works and that he is familiar with the system. (S60)

Despite perceived limitations such as the lack of transferability of statistical values to individual cases, some patients draw an analogy so far that the AI-CDSS competes with the professional as a second opinion. This point addresses the subjective feeling of patients by assuming a collegial equality between the CDSS and a professional. This equation of the AI-CDSS with a person who they have to trust due to the setting leads to a lack of trust for some patients:

I really don’t know whether I would trust this app, which really has a lot of data, or my doctor, who perhaps knows [the situation] from a patient [and] who has already had the experience—and who then tells me something else that I should do. I would be completely lost. […] I think that would simply violate my trust in BOTH, so to speak. (N79)

Due to this, the patient expresses increased indecision (S92) and states indecisiveness about whom to trust when assessments by professionals and AI-CDSS contradict each other (N79). One person emphasizes that this indecision would lead to a conflict of loyalty between patients and their doctors: “So at the time when I’m being treated by both, let's say, I don’t know who I would trust more. […] I would be totally confused as a patient” (N77).

While this behavior is somewhat reminiscent of blind trust,^49^ patients criticize that professionals could rely too much on the AI-CDSS and are no longer able to question the system's results. They see this as a risk of a possible loss of competence, knowledge, skills or gut feelings (S124, N87), and therefore emphasize that specialists should only use such AI-CDSS as support:

However, I think that the benefits [of an AI-CDSS] should be taken with a pinch of salt, because especially with inexperienced surgeons and with relatively difficult procedures, a surgeon who is not yet very familiar with it may say, “ow, I have the device, that will help me,” and then make a mistake afterwards because he relies on the device and doesn’t have the additional experience that might be necessary for that operation. (S37)

In summary, patients cite possible disagreements between the professionals and the CDSS, the professionals’ excessive or even blind trust in the CDSS, and the loss of skills through the use of the CDSS as possible reasons for mistrust.

### Responsibility

Responsibility is primarily associated with decision-making and the subject of responsibility, leading to the responsible agent: “who has my life in his hands or me in his hands” (S168), and the agent's control over the action itself, “responsibility remains with the one who is holding the knife in his hands, who guides the knife” (S150).

As stated in the trust section, respondents see technologies mainly as a tool for healthcare professionals and differentiate accordingly between humans and machines. Consequently, AI-CDSS are perceived as instruments, tools, aids, or supplements for healthcare professionals to provide support or relief, for example through additional information.

A transfer of responsibility to AI-CDSS is seen as particularly critical and dangerous. One patient refers to limitations of AI in adapting to real-world scenarios: “well, there are many different scenarios that could happen in everyday life” (H114). Others indicate a gradual projection of responsibility to AI-CDSS: “if he [the doctor] is not also tempted by such app to follow and not to look through everything again himself” (N20).

Patients see sole reliance on the results provided by the AI-CDSS negatively, particularly, unreflected reliance and a potential loss of competence and communication among healthcare professionals (H110).

Interviewees propose that the healthcare professional's task is to evaluate the tool and to verify their results: “The app offers him literature, the app offers him predictions. This has to be initially adjusted with the reality and with his patients” (S143). Additionally, patients associate responsibility of healthcare professionals with the task of acting as a contact for patients. Although producers, developers, IT specialists and hospitals are mentioned as further subjects of responsibility, their attribution of responsibility is viewed critically because of the impossibility of holding companies accountable due to the lack of contact opportunities and the burden of proof:

Yes, I see the problem primarily in the fact that the producer may potentially … in other words, that the patients bear the burden of proof. First, we would need to demonstrate that the device acted incorrectly […] How, then, can this person provide the necessary evidence? (H112, also S159, S147)

Thus, individual attribution of responsibility to producers is only presented in a limited form and only in the context of software errors.

What is particularly important to me is that as soon as the component [the CDSS] finds its place in healthcare, the question [of liability] is solved in advance, ensuring that the sole responsibility remains with the surgeon and that the patient does not subsequently have to approach the producers of the technology regarding any claims for damages. (S140, also S161, N130)

Consequently, patients express a desire to transfer responsibility to those who act directly on them, knowing this will be difficult to implement in practice (S150). Nevertheless, the responsibility should stay with those who have “the last word. He [the surgeon] gets suggestions from the artificial intelligence, but no instructions. And in this respect, the surgeon has the decision and whoever has the decision has the responsibility” (S153). This individual attribution of responsibility leads one respondent to doubt the assumption of responsibility by healthcare professionals due to their own risk (S148). While reference is made to the difficulty of refusing the AI-CDSS use due to economic pressures of the healthcare system, “because […] the clinicians and the clinic administrators, the businesspeople, who are in the clinic, they also want that the clinic has a good reputation” (S174).

Individual responsibility as a personal responsibility of the patients is stated in the context of noncompliance with corresponding therapies. It is noticeable that a lack of shared decision-making is mentioned in individual cases and that there is a desire for “that I myself am also integrated into the treatment plan. […] I could also participate actively on the therapy and would not be so left out and I would not have to say: ʻ I okay, the doctor does everything'” (N30).

Shared attribution of responsibility is mentioned in the context of the healthcare team, “an operation is teamwork” (S96) and in learning situations: “But the accompanying experienced surgeon or people, who are to be trained, should also be able to see and learn and think along and also have the information available” (S91).

Finally, patients’ view of a potential loss of theoretical and practical competence due to the unreflected and increasing use of AI-CDSS leads to questioning any assumption of responsibility: “So at work I would never anyhow hand over anything, what I cannot understand, yes? So I do think that it is totally important, that the doctor knows, based on which data the app came to its decision” (N110). The increasing loss of practical experience due to reliance and the absence of any initial experience (S37, S65) are seen as challenges with potential long-term impacts on professionals (S119).

In summary, patients primarily associate responsibility with an individual, particularly the professionals, and occasionally a team of professionals. This is especially relevant because the responsible subject also serves as a point of contact, with whom patients communicate closely and whom they can reach out to in critical situations.

### Self-determination

Patients mainly address self-determination in relation to acting on their preferences. This seems to be a major challenge of AI-CDSS application given individual limits and situational circumstances. “I believe this is questioned or torpedoed by the system, yes. I believe this is not possible anymore, this individual, self-determined setting of limits through the systems” (H106). Especially home care patients see the potential of hindering those individual processes as critical. “But simply this self-determined, this self [deciding] somewhere […] and not just the machine says: ʻ som has to be this and that now.' So, I would find this also to be very important, right?” (H125) Other respondents see limitations of self-determination, e.g., in relation to special medical situations such as unconsciousness during surgery (S122) or in emergencies (S57).

From the patients’ perspective, the respect for self-determination includes informational self-determination, e.g., highly sensitive personal health information. The misuse of such sensitive personal health information, like diagnostic results, could potentially lead to prejudice and discrimination, if for instance someone would apply to a job: “And precisely these psychological reports can have sometimes devastating effects, if they are really brought to light, if there is a leak, then we don’t even need to start and try to apply [for a job], right?” (N65). By focusing the AI-CDSS design solely on healthcare professionals as users without considering patients’ interests, patients fear they may lose their informational rights, leading to the access of data that patients do not want to allow (H58, N61).

For patients, self-determination includes the right not to know. In regard to algorithms that could predict serious illnesses or even the end of life, patients worry that these could contribute to additional burdens:

That is why I think I don’t always want to know whether I will have diabetes type II in 5 or 10 years, if I could always retreat my stats. […] So, this is just not good news and I think to myself, then I would rather not know. (N30)

Respondents discussed how such information should be communicated: “So a doctor may be able to verbally intercept this better than the app could do” (N34).

Similar concerns are evident in the home care focus group, who are strongly dependent on interpersonal care situations and skeptical of individualization through AI-CDSS. Rather, respondents see risks for dehumanization and objectification: Patients would possibly only be ventilated “according to the standard and scheme” (H110) and “no longer be taken seriously” (H170).

There is concern that patients will not anymore be perceived as communication partners:

We want professionals to communicate with us, maybe in a different manner at that moment, but we don’t want them to just […] I mean, I don’t even want to compare it to an animal. But that they just feed us and then it's enough. (H101)

This includes the ability to communicate verbally and non-verbally, and to clarify and explain AI-CDSS technology. This may be difficult due to a lack of “science communication” (N144) or a lack of inclusion in the informational transparency of decision-making processes (N75). It is obvious from the patients’ perspective that the rational understanding of such highly complex technological processes is a challenge for self-determination. “It is a thin line. […] So, I also think that a good ethical, moral standard for behavior needs to be developed, so that it [the device] can be handled well for all sides” (H135).

The importance of self-determination is reflected in the interviewees’ statements on the patient's will, which is seen as “the highest good that we have, even if we can no longer express it ourselves” (H70), and which should not be ignored under any circumstances (H78). Any wishes could cover decisions for or against the use of technology, including the consequences:

So they simply did it with a hand gesture, because they could no longer speak […] made it clear with a wave of the hand that they no longer wanted the mask, right? And you have to respect that, because it was probably written somewhere as their last will. (H139)

However, asserting a patient's will can mean self-determination against “reasonable” options in medical care. The right to act irrationally is perceived as essential, most clearly in the home care focus group.

We also have a right to medical irrationality and this is of course completely undermined by such a device. If the device says: “You are being overbreathed, you are getting far too much air” […] and the person themselves says: “[…], I know, but I need this, because I can speak better this way.” Then there is already a conflict: Am I allowed to act against the medical advice of this device? That is also a conflict for the caretaker. “Okay, do I follow, what the patient says or what the device says?” (H 121)

Other focus groups are also critical of the challenge of compliance with regard to human behavior: “Nobody admits to the doctor that they permanently mess it up” (N26), though for some, technology has the advantage to control their own participation (N24).

While the last aspect indicates that respondents place particular value on the role of personal responsibility, patients also detect a loss of active involvement in their own diagnosis and treatment process. This could be counteracted by participating in AI-CDSS. The patient could be “the proper partner in treatment” (N95), who is motivated by involvement “to enter and measure such things” (N34). “On the other hand, it would of course be better or even more constructive; if from both sides [data] could be fed in” (N28) to avoid single perspective diagnoses.

In summary, patients value highly being seen as active communication partners in the course of action and decision-making process, whereby respect for the patient's will and preferences is particularly emphasized. This includes informational self-determination, which not only encompasses the handling of highly sensitive personal health information, but also the right not to know.

## Discussion

**Figure 1.**
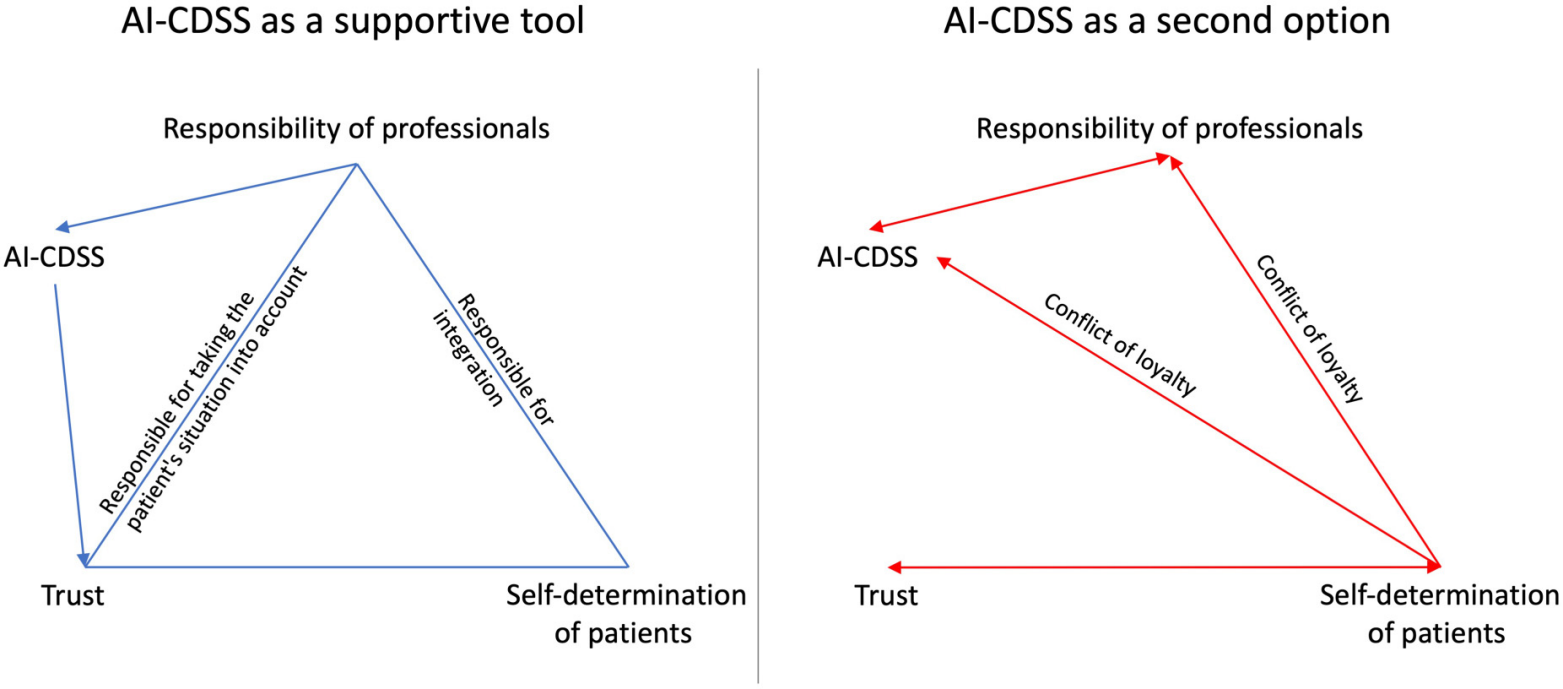
Perception of the AI-CDSS from the patients’ perspective and its effects on the aspects of trust, responsibility and self-determination within the relationship between patients and healthcare professionals as a supportive tool (blue, left side) and a second opinion (red, right side) (own illustration).

The focus of this study is on patients’ views of integrating AI-CDSS into routine care. One of the main findings illustrates that from a patient's perspective, a trusting relationship between healthcare professionals and patients is essential for the AI-CDSS use. Responsibility, trust, and self-determination seem to be very closely interrelated and, more or less, in balance; they are partly mutually dependent (see Figure 1, blue) but can also cancel each other out (see Figure 1, red). Our results demonstrate that trust and responsibility are based on some similar prerequisites: patients not only emphasize the necessary technical expertise of healthcare professionals (i.e., the availability of specialist knowledge) as a precondition for trust and responsibility. They further refer to the necessary empirical knowledge and the ability to reconcile the specific situation of the patient with the standards of professional knowledge.

Responsibility and trust can therefore only be comprehensively attributed if knowledge of the structure, application and experience of the AI-CDSS can be applied to individual cases by the professional. Due to the assumptions made, healthcare professionals become subjects of responsibility in a decision-making process that could face significant challenges in the future. Physicians, for example, would have to develop AI literacy in ongoing work processes, which would increase workload and could possibly lead to a reconsideration of the application altogether.^13,50^ The question remains as to the necessary extent of required knowledge of AI, especially concerning technical aspects.^51^

Furthermore, responsibility is deeply connected with the assumption and fulfillment of tasks that contribute to a good diagnostic and therapeutic process for patients. With regard to the AI-CDSS application, this concerns the (design) development and maintenance. These qualities play a decisive role with regard to responsibility, as patients see healthcare professionals as being responsible for successful interpersonal communication, even in cases of technical malfunctioning of the AI-CDSS. This correlates to them being the contact person for patients. Patients emphasize the importance of being perceived as a person^14^ and of being considered in their specific situation by their healthcare professionals.^52^ Hence, they wish for their counterpart to be a competent contact person. However, they also expect healthcare professionals to involve them as experts in their situation within a shared decision-making (cf. Figure 1). This expectation and the resulting distribution of responsibilities and tasks can then lead to a basis of trust, because trust only seems possible in interpersonal relationships through the transparency of the respective tasks and instructions. Yet, patients appear to be particularly reliant on trust in healthcare professionals when their self-determination is restricted, leading to the third aspect of self-determination. Accordingly, the self-determined approach of patients towards responsibility and trust changes according to the tasks assigned to the healthcare professionals or AI-CDSS, which leads to challenges for the patients.

As soon as AI-CDSS are implemented into the treatment context, healthcare professionals are not only given responsibility for aspects of the treatment situation, but also for the AI-CDSS integration into the treatment context. Overall, patients are ambivalent about the AI-CDSS use on the relationship of trust between healthcare professionals and patients. Their assessment fluctuates between monitoring and control, with the technical expertise of the professionals playing a key role in their assessment:

Firstly, AI-CDSS can be perceived as a supportive tool in the treatment context. The integration is perceived as an extension of the expertise of the professional and a supplement to their knowledge and experience. In this case, trust in the system is mostly a proxy for trust in the healthcare professionals and their judgment. Ultimately, this means that decisions in the treatment context can (continue to) be made in shared decision-making processes. Patients do not see the AI-CDSS involvement as a threat, but as a useful addition to achieve better results. Hummel et al. argue in a similar direction when they emphasize that a lack of specialist knowledge on the part of professionals could be compensated by data-intensive AI-CDSS under the right conditions.^52^ However, it means that healthcare professionals are more likely to be held responsible by patients for incorrect predictions made by AI-CDSS, although patients are aware that healthcare professionals can hardly bear the responsibility.

Secondly, AI-CDSS can be perceived as a second opinion. In this case, specialist knowledge is attributed primarily to the AI-CDSS and less to the professionals; the latter are often only credited with knowledge of the specific patient situation, if at all. This is quite contrary to the patients’ demands that professionals have the sole responsibility because they are given the task of subsuming “the particular case under the generality of the evidence base.”^52^ Perceiving an AI-CDSS as a second opinion means that the professional expertise of the healthcare professional may be called into question—especially if the professional's judgment differs from the analysis or recommendation of the AI-CDSS. Similar to what Kerasidou has pointed out,^53^ there could be a paradigm shift within the healthcare system, with trustworthiness increasingly associated with greater accuracy and effectiveness of AI-CDSS rather than relational values such as human trust.^14^ Patients react to this with indecision and, in cases of doubt, are confronted with a conflict of loyalty and loss of trust.

While the trusting relationship is the focus of the patients in the outlined perspectives on AI-CDSS, conclusions can also be drawn about the aspects of responsibility and self-determination. A trusting relationship develops for the patients interviewed above all when they feel perceived as equal actors in the healthcare-patient setting. However, patients do not see the aspect of trust as exclusively positive, but rather as a necessity, for example, if they have the feeling of being at the mercy of healthcare professionals.

Patients have the impression that it could be difficult to maintain their self-determination if AI-CDSS are used. They argue that they want to take on more personal responsibility within the therapy, for example, by taking on disease management independently. In particular, patients see the risk that the AI-CDSS integration could lead to the loss of at least one actor in the healthcare-patient setting, which contradicts previous assumptions that their integration will lead to a “doctor-computer-patient relationship.”^54,55^

[1] The loss of one actor in the relationship: Patients fear that professionals might trust AI-CDSS to a very high degree and may even become dependent on them.^52^ As a result, patients may find themselves negotiating with the AI-CDSS on their own, as the professional withdraws from the conversation with the patient. Although patients do not have the necessary expertise to subsume their specific situation and choose the best option for them, patients would have to bear a high degree of personal responsibility. The opposite effect occurs when little importance is attached to the patient's perspective and preference. The new form of paternalism in the sense of “doctor knows best—but the computer knows more and makes fewer mistakes”^14^ would make shared decision-making processes virtually impossible.
[2] The loss of two actors in the relationship: If, conversely, AI-CDSS are perceived as the deciding factor, i.e., AI-CDSS are perceived as the only option, then both patients’ preferences, e.g., the right to medical irrationality that they demand, and professional expertise could become obsolete.^56–58^ In contrast to the case outlined above, none of the human actors are seen as responsible anymore, because the AI-CDSS becomes the decision-maker. As Banerjee et al. outline, “trust in algorithms cannot be completely decoupled from trust in institutions and people involved in the research.”^59^

The fact that patients consider it likely that AI-CDSS will be used both as a support tool and as a second opinion can be interpreted as a sign of their own uncertainty and indicates that the social role of the AI-CDSS in professional-patient relationship has not yet been sufficiently clarified.^60^ Depending on the social role that patients ascribe to AI-CDSS, the involvement of AI-CDSS may disrupt the provider-patient relationship, and negatively impact their relationship of trust. At the same time, the increasing complexity within the healthcare system may collide with patients’ need for transparent structures. Additionally, to the previous recommendations to emphasize the supporting function of AI-CDSS and to call for adjustments to medical training,^14^ it appears necessary to empower all stakeholders involved in the decision-making process with regard to data literacy, especially patients.

## Limitations

The study has some limitations that are characteristic of qualitative studies with an exploratory approach. First, the participants were recruited through self-help groups, which tend to be more interested and involved in their care process and related topics than other patients. This selection bias should be noted with regard to the thematic focus, as the topic may have particularly appealed to patients who are interested in digital health. Since we were unable to conduct analog focus groups due to the COVID-19 pandemic and the contact restrictions prevailing at the time, we decided to use online focus groups to explore the patients’ perspective on the use of AI-CDSS. We were aware that online formats are often perceived as less engaging and that the technical format could exclude some people from participation^61^ but we had to accept these risks in the absence of alternatives. Our sample shows that we succeeded in recruiting people of all age groups with our recruitment strategy. However, the age of the subjects was not a relevant characteristic for us in our selection criteria; instead, we focused on disease-specific patient groups.

Second, the case studies were limited due to their selected medical specialties. Hence, they represent only a subset of possible implementations. It is important to note that the selected case vignettes also differ in terms of the prerequisites for the use of AI-CDSS or in the way professionals interact with patients, e.g., AI-CDSS are already used in isolated cases in surgery, while our case vignette for home care is much more demanding. These application-specific characteristics were taken into account as far as possible when evaluating the results. The exploratory study used hypothetical CDSS case vignettes as stimuli to be evaluated by the participants, who had no experience with AI-CDSS in their medical history. Therefore, there may have been challenges regarding the translation of case reality and anticipated reality. Whether these anticipated realities and their challenges will exist in the future, cannot be stated.^62^

## Conclusion

The AI-CDSS implementation may offer various advantages for patients and healthcare professionals such as efficiency, support or just as a back-up, but may also cause challenges. The latter is particularly evident in the aspects of trust, responsibility, and self-determination, which interact with each other. Patients might have a very different perspective on these technologies, due to their interests, values and educational backgrounds. Therefore, AI-CDSS development and implementation processes need to consider all potential users of these systems, including patients. Our exploratory study is one of the first empirical studies to focus primarily on the patients’ perspective and to explore their view on potential changes in the provider-patient relationship with regard to trust, responsibility and self-determination. We concentrated on selected ethical aspects to obtain a more comprehensive picture of patients’ attitudes and acceptance of AI technology.

Patients indicate that their interaction with AI-CDSS can lead to challenges regarding trust, responsibility, and self-determination and that these aspects might be lost. Understanding AI-CDSS as both supportive tools and second opinions, emphasizes these findings. While there is already an established discourse on the implications of attributing AI-CDSS as supportive tools, which refers to aspects of the responsibility debate, the discourse on AI-CDSS, to which the social role of second opinion is attributed, is not yet an extensive part of current debates. For example, it is unclear how the stakeholders, i.e., providers and patients, could deal with the conflict of loyalty described by patients if AI-CDSS are ascribed more professional competence than the attending healthcare professionals. There is also no significant discourse on the challenge of ensuring that the patient's perspective is guaranteed as an elementary part of shared decision-making processes, even with the usage of AI-CDSS. Against this backdrop, trust in professionals is an essential aspect of the healthcare environment, provided that AI-CDSS are understood as supporting tools for professionals and do not represent second opinions of their own.

Our findings point to dynamics of trust, responsibility and self-determination that are already emerging today and to effects that may emerge in the relationship between healthcare professionals and patients in the future. Therefore, our results suggest that patients should be more involved in the development and implementation process of AI-CDSS to gain a deeper understanding of individual decision-making processes and to ensure that their interests are better taken into account. Furthermore, the results indicate that healthcare professionals need to be empowered to inform patients about the functions and possibilities of AI-CDSS in healthcare. In conclusion, the study contributed to a better understanding of the deliberation and decision-making processes of patients in relation to the use of AI-CDSS.

## Supplemental Material

sj-docx-1-dhj-10.1177_20552076251339522 - Supplemental material for Indecision on the use of artificial intelligence in healthcare—A qualitative study of patient perspectives on trust, responsibility and self-determination using AI-CDSS Supplemental material, sj-docx-1-dhj-10.1177_20552076251339522 for Indecision on the use of artificial intelligence in healthcare—A qualitative study of patient perspectives on trust, responsibility and self-determination using AI-CDSS by Diana Schneider, Wenke Liedtke, Andrea Diana Klausen, Myriam Lipprandt, Florian Funer, Tanja Bratan, Nils B. Heyen, Heike Aichinger and Martin Langanke in DIGITAL HEALTH

sj-docx-2-dhj-10.1177_20552076251339522 - Supplemental material for Indecision on the use of artificial intelligence in healthcare—A qualitative study of patient perspectives on trust, responsibility and self-determination using AI-CDSS Supplemental material, sj-docx-2-dhj-10.1177_20552076251339522 for Indecision on the use of artificial intelligence in healthcare—A qualitative study of patient perspectives on trust, responsibility and self-determination using AI-CDSS by Diana Schneider, Wenke Liedtke, Andrea Diana Klausen, Myriam Lipprandt, Florian Funer, Tanja Bratan, Nils B. Heyen, Heike Aichinger and Martin Langanke in DIGITAL HEALTH
