# Supplementary material for: Indecision on the use of artificial intelligence in healthcare—A qualitative study of patient perspectives on trust, responsibility and self-determination using AI-CDSS
Source: Digit Health. 2025 May 30;11:20552076251339522. doi: 10.1177/20552076251339522 (PMC12134509; doi:10.1177/20552076251339522)
Supplement: sj-docx-1-dhj-10.1177_20552076251339522 - Supplemental material for Indecision on the use of artificial intelligence in healthcare—A qualitative study of patient perspectives on trust, responsibility and self-determination using AI-CDSS [file sj-docx-1-dhj-10.1177_20552076251339522.docx]

**Supplement**

Interview guides was first published in Funer, Schneider, Heyen et al. 2023 and is presented in abridged form.

| **Guiding question(s)** | **Aspects to be addressed/ concrete demands** | **Maintenance issues/ control issues** |
| --- | --- | --- |
| Technique check [half an hour before starting the focus group] | | |
| - First participants appear - Informal greeting - Test: microphone, camera, raise/lower hand, mute   Possibility to customize displayed name | | |
| Topic complex: Entry [start of the focus group] | | |
| - Welcome and introduction of the moderator [NH, HA, TB] and reference to DS [protocol and back-up moderation] - Background of the DESIREE project: From an ethical and social perspective, we want to explore the effects of clinical decision support systems (in the form of digital tools or apps) in medicine and care, e.g. in relation to trust in nursing and medical action or in relation to the doctor-patient relationship. We are particularly interested in the opinions and views of patients.     Before we begin...   - Explanation of technology: camera on, microphone off [details probably unnecessary because everything was checked beforehand] - Renewed information of the group discussion:   1. We would like to record the conversation so that we can evaluate it more easily. We only make a sound recording, without a picture. When transcribed, the conversation is pseudonymised, as described in the information sheet.   2. Your participation in this group discussion is voluntary. You can cancel or interrupt the discussion at any time.   3. Some of you may know each other and others may not. For today, everything that you report here in this protected (virtual) room about yourself, your experiences, what you would like to talk about stays "in this room". This means that no information about group members may be leaked to people outside of this (virtual) space. The keyword here is confidentiality.   4. It is of great interest to us to hear your arguments, perspectives and associations on the topics discussed. Please do not hesitate to express your own opinion. We are not concerned with finding a common consensus here and now. There is also no right or wrong opinion. We are much more interested in your individual and different thoughts and experiences. These are very important for our research.   5. We want to have a group discussion. This means - unlike what you may know from classic interviews - that we don't ask you a lot of questions and you then answer us. Rather, we have specific topics that interest us and we'd like to hear your thoughts on them. We would therefore like to invite you to talk to each other and exchange views on these topics. We will accompany this conversation. My colleague, [DS], will hold back and observe the discussion. I myself will also ask you one or two questions.   6. We're welcome to take a short break in between, I'll suggest that after about 1 hour. If you need a break beforehand, please let us know.   7. Addressing Each Other: I suggest we address each other by the names as they appear here, if you agree? You can also change your name display.     We will also put these 7 points in short form in the group chat [DS: put 7 points in the chat]. Do you have any questions about these points?   - 1. The conversation is recorded (audio only).   2. Participation is voluntary. The conversation can be stopped or interrupted at any time. | | |

| 1. The conversation is confidential. No information about group members is given to people outside of this (virtual) space. 2. Everyone is free and open to express their opinions and thoughts. There are no right or wrong statements. 3. The moderator will tend to stay away from the conversation and only ask questions from time to time. 4. We take a break after about 1 hour (as needed). 5. We use the names shown here.     We'll turn on the recorder now. | | |
| --- | --- | --- |
| Introduction round of participants | | |
| Please introduce yourself...  [DS begins; the next participant is invited to speak by passing on a virtual pen] | - full name or first name or pseudonym - Background information on your own illness - Type of membership in the self-help group - Getting to know each other |  |
| Introduction of case vignettes [15 minutes after starting the focus group] | | |
| *Presentation of case vignette* (depending on the self-help group)    [moderator shares screen (200%) and reads out; Give 1-2 minutes extra time, stop on picture at the end] | • Was the text easy to understand or did something important remain unclear? |  |
| Topic complex 1: trust and acceptance [30 minutes after starting the focus group] | | |
| **Key question 1 (spontaneous reaction)**    What comes to your mind in this scenario?  [ensure that each participant has commented on this opening question] | - (Does this type of treatment appeal to you or not?) - Are there certain expectations or fears that you associate with it? | Can be used for all subject areas:     - Can you describe that in more detail? - Tell me a little bit more about it. - Can you tell/describe … in a little more detail? - Can you give an example of...? - Did that... matter? - Mr/ Ms [name x] said this, Ms/ Mr [name y] said that. What was it like with the other people? - I notice that you [describe the feeling]. - Provocation: Aha, and that's exactly not the case here?   What's different here? |
| **Key question 2 (advantages/disadvantages)**    If you, like Ms. Müller/ Mr. Müller, are asked whether you agree to the use of the clinical decision support system (CDSS): Under what conditions could you imagine that such a CDSS would be used as part of your treatment?    [End Screen Sharing] | - Under what conditions would you consider [field of   application] using the  CDSS?   - When and under what circumstances should the CDSS not be used? - What specific expectations or fears would you have?     [If not raised in the discussion, follow up; towards the importance of aspects such as misinformation, data protection, privacy, biases in the data; the fact that a CDSS makes recommendations:] |  |

|  | - Does it matter to you that a CDSS knows so much about you and/or constantly monitors you? Are there things that you would prefer not to be captured by the CDSS? What would that be and why this particular data? - Are there perhaps still aspects that do not concern you directly, but which you consider relevant in this context? |  |
| --- | --- | --- |
| Topic complex 2: Professional-patient relationship and responsibility [one hour after starting the focus group – before that, if necessary, a short break of 5-10 min] | | |
| **Key question 3 (relationship,**  **trust, traceability, responsibility)**    Would involving the CDSS change your relationship with your professional in any way? | Which aspects could or what could possibly change?    How would you have different expectations of your professional when using a CDSS?   - What difference would it make to you if your professional was supported by such a CDSS? - How important would it be for you to know when the CDSS is in use (and when it is not)? - Does a professional seem more competent or rather less competent if you know that she/he can be supported digitally? - How could your trust in your professional change?     *Responsibility*  Imagine if there was a treatment error in your case: Does it make a difference to you that your professional was supported by such a system? Who would you blame for the mistake? • If necessary, follow up: Does [the person or entity that made the fundamental decision to use the CDSS, approval, developers?] also play a role?   - If the CDSS makes a recommendation that the professional doesn't think is reasonable, what should the professional do? |  |
| Case-specific introduction to the question: |  |  |
| Home care:   - If you now imagine that your professional - as in the example of Ms. Müller - would be helped by the CDSS in the event of an alarm: [above question].   Nephrology:   - Mr. Müller had the impression that the treatment discussion was as usual. How about you? If the CDSS were used in your treatment context, [above question].   Surgery:   - If you now imagine that your surgeon would be supported by such a CDSS during the operation, as in the example of Ms. Müller:   [question above]. |  |  |

|  | *Explainability of decisions*  From the patient's point of view: How important is it for you to be able to understand why certain decisions were made in an emergency situation, for example why it was checked first whether the tube was disconnected before other possible error sources were checked?    From the professional’s point of view: If you know on the one hand that patient safety has increased overall with the help of  artificial intelligence, but that the individual recommendation for a specific troubleshooting measure is not necessarily comprehensible for the professional, what does that mean for you/what do you think personally about it?    *If necessary: Right to know/not to know [only for Nephro case vignette]*  Imagine that in your case the CDSS makes a prediction about a short remaining lifetime. Even if the CDSS is not intended to share such forecasts with patients - would you want to know or not? |  |
| --- | --- | --- |
| Additional questions (optional) [105 minutes after starting the focus group] | | |
| What should future generations of professionals definitely be able to do, i.e. have learned in their training, if you imagine a future in which such CDSS are increasingly used? | Which skills are perhaps not so important anymore? |  |
| Ending | | |
| - Now that we have talked in detail about the use of the CDSS: Has anything changed from your initial opinion? - Is there anything else you'd like to share that we haven't addressed yet? | If you had the choice: Would you decide for or against using such a system? |  |
| Farewell [115 minutes after starting the focus group] | | |
| - Note of thanks - Remind again about confidentiality | | |
| • Participants will receive an expense allowance of 20 € (we will send a form afterwards) | | |
| Socio-demografic Check-list (asked via email) | | |
| 1. Gender    1. masculine    2. female    3. diverse 2. May I ask your age? 3. What is your role today?    1. patient    2. relative 4. What is your highest level of education? 5. What is your professional background? 6. Are you currently employed? | | |

# Case vignette Home Ventilation Care: Presentation of the “Safety box”

Ms. Müller is 54 years old and suffers from COPD, chronic obstructive bronchitis. Two years ago she contracted pneumonia and she had to be invasively ventilated in hospital. Subsequent weaning was not possible, since she had been invasively ventilated via the trachea. She lives in a house with her working husband and is looked after by a 24-hour intensive care service.

The care service in question uses a decision support system connected to the ventilator. This is based on machine learning (ML) methods, which are assigned to artificial intelligence (AI). This allows the system to quickly identify life-threatening situations, such as the hose becoming detached or the cannula clogging up and helps to resolve them. To do this, it evaluates information from the ventilator, such as breathing rate, oxygen saturation, blood pressure and heart rate. In addition, a thermal imaging camera with a motion sensor is installed above Ms. Müller's bed, which records whether Ms. Müller is alone or whether there is another person in the room. The vital values for the condition of the lungs and heart are displayed on a monitor, the system also offers information on the care activities carried out and shows how long a critical condition such as low oxygen saturation or the detachment of the tube has lasted. The various pieces of information are collated and evaluated by the decision support system, enabling the system to give caregivers and relatives a clear recommendation on what to do in an emergency situation.

There was such a situation this morning: Intensive care nurse Elena suctioned Ms. Müller during her duty and removed the ventilation tube. After the successful suction, she reconnected the ventilation tube to Ms. Müller's tracheostomy and then cleaned it up. While disposing of the rubbish in front of the house, she meets one of the Müllers' neighbors and talks to him. However, the breathing tube was not properly connected and detaches itself without this being apparent. The decision support system recognizes the situation and triggers an alarm, whereupon Elena rushes to Ms. Müller's side. The following recommendation is shown on the display: "Please check the connection to the ventilation hose". This way, Elena knows immediately what needs to be done and can quickly reconnect the hose to the ventilator so that Ms. Müller is not harmed. Without this clear instruction, she would probably have searched longer for the cause of the problem.

# Case vignette Nephrology

Mr. Müller is 54 years old and suffers from a chronic kidney disease. He is familiar with the regular visits and examinations by his nephrologist. When he arrives at the practice this time, he is presented with a new information sheet. It informs him that the nephrologist is now using a digital clinical decision support system in the form of an app in her practice. This is based on machine learning (ML) methods, which are assigned to artificial intelligence (AI).

According to the information sheet, which also includes the figure below, the app contributes to improved and personalized patient care. Since chronic kidney disease is a very complex clinical picture, the app is intended to support the nephrologist in evaluating and integrating the large amount of data relevant to his treatment. These include clinical parameters, biomarkers, medication, medical complications, lifestyle and medical history. At the heart of the app are mathematical models for personalized diagnosis refinement and for predicting adverse medical events (e.g. heart attack, stroke or terminal kidney failure) and individual disease progressions.

On this basis, the app points out possible contradictions in the data, can reduce potential prediction errors and improve medical therapy recommendations. For example, the nephrologist has the opportunity to change patient parameters (medication, lifestyle, etc.) virtually within the app and to simulate the resulting development of the disease. This helps her to assess how much a change in lifestyle influences a certain risk of disease. The nephrologist always retains full control over all medical decisionmaking processes.

Mr. Müller agrees that his nephrologist uses this app and is curious whether the treatment will change as a result. As always, a blood sample is taken and then he has to sit in the waiting room for a while until the results of the bloodtest are evaluated. Then he is called into the treatment room. Looking at her computer, the nephrologist explains the current findings and advocates continuing with the current treatment. That is the best thing for him, so his kidneys function for as long as possible. Shortly afterwards, when Mr. Müller is on his way home, he remembers the information sheet he signed today. He wonders if the nephrologist actually used this new app. He himself has the impression that the treatment discussion went as usual.

# Case vignette Surgery

Ms. Müller is 58 years old and suffers from rectal cancer. A combination of radiotherapy and chemotherapy reduced the size of the tumor but did not make it disappear. The tumor is now to be surgically removed. Due to the location and size of the tumor, however, there is an increased risk that the sphincter function will be impaired after the operation and that Ms. Müller could suffer from incontinence problems.

Ms. Müller knows that it is a very difficult operation that depends very much on the experience of the surgeon. She is worried and of course hopes for a good surgical result with as few long-term restrictions as possible. The operation should be performed in a renowned tumor center. The attending surgeon explains to Ms. Müller the various options. She tells her that a minimally invasive operation using the "keyhole technique" will be performed. In the tumor center there is the possibility that the surgeon can be supported during the operation by an "OP navigation system".

This system uses computer-aided calculations and artificial intelligence to combine the live images recorded during the operation with other diverse information from Ms. Müller's medical record, e.g. with MRI and CT images from Ms. Müller's preliminary examinations.

During the operation, the surgeon sees the live images through data glasses and is also shown additional information that can be projected directly onto the area to be operated on. For example, the system can label the tumor and different organs and structures and display them in different colors. This results in a much clearer situation, which can be a good support, especially for less experienced surgeons.

The system also suggests an incision in which the tumor should be completely removed and healthy tissue should be damaged as little as possible, or it warns of an impending injury, e.g. if nerves or blood vessels could be damaged. The system should support the surgeon in her work, but not replace her. However, even for a technical system, it cannot be completely ruled out that in individual cases structures are incorrectly recognized and a displayed recommendation could damage Ms. Müller. The surgeon must therefore decide individually whether to follow the displayed recommendation or not.

The surgeon asks Ms. Müller whether she agrees to the system being used in her surgery.
