# Supplementary material for: Indecision on the use of artificial intelligence in healthcare—A qualitative study of patient perspectives on trust, responsibility and self-determination using AI-CDSS
Source: Digit Health. 2025 May 30;11:20552076251339522. doi: 10.1177/20552076251339522 (PMC12134509; doi:10.1177/20552076251339522)
Supplement: sj-docx-2-dhj-10.1177_20552076251339522 - Supplemental material for Indecision on the use of artificial intelligence in healthcare—A qualitative study of patient perspectives on trust, responsibility and self-determination using AI-CDSS [file sj-docx-2-dhj-10.1177_20552076251339522.docx]

**Consolidated criteria for reporting qualitative studies (COREQ)**

| No | Item | Guide description | Reported on Page |
| --- | --- | --- | --- |
| DOMAIN 1: RESEARCH TEAM AND REFLEXIVITY | | |  |
| Personal characteristics | | |  |
| 1 | Interviewer/ facilitator | *Which author/s conducted the focus group?*  The focus groups were conducted by the authors TB, NBH, HA, ADK, and DS. DS was present in all focus groups and acted as back-up moderator. | *p.14* |
| 2 | Credentials | *What were the researcher’s credentials? E.g. PhD, MD*  Apart from DS and ADK, all researchers have a doctorate. DS is a PhD student. | *p.4* |
| 3 | Occupation | *What was their occupation at the time of the study?*  ML held a professorship. All the other researchers were employed as junior or senior researchers by their respective research institution at the time of the study. | *p.4* |
| 4 | Gender | *Was the researcher male or female?*  The team consisted of three men and six women. | *p.4* |
| 5 | Experience and training | *What experience or training did the researcher have?*  Researchers who were responsible for conducting the empirical study are trained in qualitative methods of social research and have many years of experience. | *p.4* |
| Relationship with participants | | |  |
| 6 | Relationship established | *Was a relationship established prior to study commencement?*  No relationship was established prior the study commencement. | *p.4* |
| 7 | Participant knowledge of the interviewer | *What did the participants know about the researcher? e.g. personal goals, reasons for doing the research*  At the beginning of the focus groups, the project team introduced themselves and explained the research objectives and purpose of the project and answered any questions the participants had about the project. | *p.4* |
| 8 | Interviewer characteristics | *What characteristics were reported about the interviewer/facilitator? e.g. Bias, assumptions, reasons and interests in the research topic*  The interviewers are not medical professionals, but are familiar with the medical system in Germany, have dealt intensively with the applications of innovations such as digitalisation and AI in healthcare in various project contexts and are sensitized to the ethical and social implications of AI in healthcare. | *p.4* |
| DOMAIN 2: STUDY DESIGN | | |  |
| Theoretical framework | | |  |
| 9 | Methodological orientation and Theory | *What methodological orientation was stated to underpin the study? e.g. grounded theory, discourse analysis, ethnography, phenomenology, content analysis*  Data analysis was performed using structured qualitative content analysis according to Kuckartz and Rädiker (2022). | *p.5* |
| Participant selection | | |  |
| 10 | Sampling | *How were participants selected? e.g. purposive, convenience, consecutive, snowball*  Convenience sampling was performed. | *p.4* |
| 11 | Method of approach | *How were participants approached? e.g. face-to-face, telephone, mail, email*  Acquisition took place with the help of self-help groups. | *p.4* |
| 12 | Sample size | *How many participants were in the study?*  We conducted three focus groups with 18 patients (5-7 per group). | *p.4* |
| 13 | Non-participation | *How many people refused to participate or dropped out? Reasons*?  Apart from one participant (acute hospitalization), all people recruited for the study took part in the focus groups. | *p.4* |
| Setting | | |  |
| 14 | Setting of data collection | *Where was the data collected? e.g. home, clinic, workplace*  Due to the COVID-19 pandemic and mobility issues of some patients, all focus groups were conducted via video call. Respondents were generally at home during the focus groups. | *p.4* |
| 15 | Presence of non-participants | *Was anyone else present besides the participants and researchers?*  Most respondents were alone unless they needed assistance of a caregiver. However, caregivers did not actively participate. | *p.4* |
| 16 | Description of sample | *What are the important characteristics of the sample? e.g. demographic data, date*  The sample consisted of 12 women and six men aged between 24 and 76. The average age was 47.8 years for the nephrology focus group, 51.8 years for the surgery focus group and 39.4 years for the home ventilated care focus group. | *p.5* |
| Data collection | | |  |
| 17 | Interview guide | *Were questions, prompts, guides provided by the authors? Was it pilot tested?*  The interview guide was open-ended but contained a number of key questions and possible prompts. The group discussions generally followed a similar approach, although the questions themselves varied depending on the participants. During the focus group, a stimulus was used which could be viewed by the respondents. | *p.4/5* |
| 18 | Repeat interviews | *Were repeat interviews carried out? If yes, how many?*  No repeat focus groups were conducted. | *No* |
| 19 | Audio/visual recording | *Did the research use audio or visual recording to collect the data?*  Audio recordings were made of the focus groups. Recordings were transcribed ad verbatim and pseudonymized. | *p.5* |
| 20 | Field notes | *Were field notes made during and/or after the interview or focus group?*  Field notes were taken during and after the focus groups. | *p.5* |
| 21 | Duration | *What was the duration of the interviews or focus group?*  The focus groups lasted an average of 114:17 min (range between 110:07 to 123:04 min), including a short break after one hour. | *p.4* |
| 22 | Data saturation | *Was data saturation discussed?*  The data collection was concluded when theoretical saturation was achieved, meaning that additional focus groups did not yield any new information regarding the research question. | *p.5* |
| 23 | Transcripts returned | *Were transcripts returned to participants for comment and/or correction?*  Transcripts were not returned to participants for comment and/or correction. | *No* |
| DOMAIN 3: ANALYSIS AND FINDINGS | | |  |
| Data analysis | | |  |
| 24 | Number of data coders | *How many data coders coded the data?*  The first authors DS and WL coded the interviews independently and discussed the individual segments, especially critical segments. | *p.5-6* |
| 25 | Description of the coding tree | *Did authors provide a description of the coding tree?*  The codes were tagged and described in the software MAXQDA 2020, unless they were self-explanatory. | *No* |
| 26 | Derivation of themes | *Were themes identified in advance or derived from the data?*  The main topics were defined in advance; details and participants' priorities on the topic of AI in healthcare were derived from the data. | *p.5* |
| 27 | Software | *What software, if applicable, was used to manage the data?*  For coding data MAXQDA 2020 was used. | *p.5* |
| 28 | Participant checking | *Did participants provide feedback on the findings?*  Participants provided no feedback on the findings. | *No* |
| Reporting | | |  |
| 29 | Quotations presented | *Were participant quotations presented to illustrate the themes / findings? Was each quotation identified? e.g. participant number*  Participant quotations were presented to illustrate the themes / findings. Each quotation is identified by the name of the focus group and the corresponding paragraph number. | *pp.6-10* |
| 30 | Data and findings consistent | *Was there consistency between the data presented and the findings?*  Consistency between the data presented and the findings exists. | *pp.6-10* |
| 31 | Clarity of major themes | *Were major themes clearly presented in the findings?*  Major themes are clearly presented in the findings. | *pp.6-10* |
| 32 | Clarity of minor themes | *Is there a description of diverse cases or discussions of minor themes?*  Minor themes are closely related to the main topics and were discussed as part of the results. | *pp.6-10* |
